# Protocol for development, visualization, and quantification of mycobacterial biofilms on primary human airway epithelial cells

**DOI:** 10.1016/j.xpro.2025.103872

**Published:** 2025-06-04

**Authors:** Amy M. Barclay, Ronald W.A.L. Limpens, Montserrat Bárcena, Tom H.M. Ottenhoff, Pieter S. Hiemstra, Anne M. van der Does, Simone A. Joosten

**Affiliations:** 1 Leiden University Center for Infectious Diseases (LUCID), Leiden University Medical Center (LUMC), 2333ZA Leiden, the Netherlands; 2 Cell and Chemical Biology (CCB), LUMC, 2333ZA Leiden, the Netherlands; 3 PulmoScience Lab, LUMC, 2333ZA Leiden, the Netherlands

**Keywords:** Immunology, Microbiology, Microscopy

## Abstract

Tuberculosis (TB) displays several characteristics commonly linked to biofilm-associated infections, including recurrence of infection and resistance to antibiotics. Studying biofilm formation by mycobacteria on relevant mucosal surfaces advances our understanding of its role in TB pathogenesis and drug tolerance. Here, we present a protocol to promote biofilm formation by mycobacteria on differentiated primary pseudostratified human bronchial epithelial cell cultures. We then detail procedures to visualize and quantify biofilm matrix and biomass using electron and confocal microscopy and crystal violet staining.

For complete details on the use and execution of this protocol, please refer to Barclay et al.^1^

## Highlights

• Instructions for assessing mycobacterial biofilm formation on a biotic surface
• Steps for visualizing biofilm matrix with electron and confocal microscopy
• Guidance on quantifying biofilm biomass and matrix components

## Abstract (editor highlights)

Publisher’s note: Undertaking any experimental protocol requires adherence to local institutional guidelines for laboratory safety and ethics.

## Graphical abstract

**Figure:**
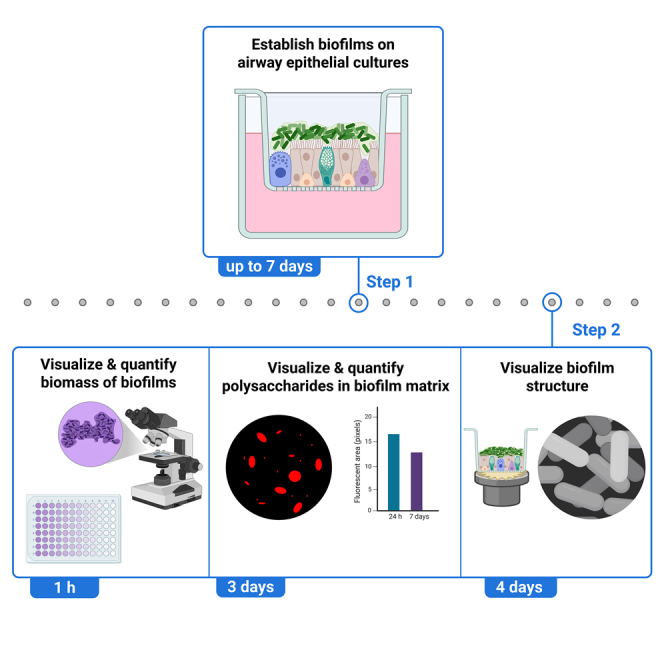

## Before you begin

Biofilm infections are notoriously difficult to eradicate and are often associated with recurrence and antibiotic resistance. Mycobacteria such as *M. tuberculosis* can also form biofilms *in vitro* and some studies suggest the presence of biofilms *in vivo* as well.^2^^,^^3^ Studying mycobacterial biofilm formation on a relevant mucosal surface such as the airway epithelium may provide insights in TB pathogenesis and improve treatment strategies. This protocol describes the steps to culture mycobacterial biofilms on air-liquid interface cultures of well-differentiated primary human bronchial epithelial cells (ALI-PBEC). Next, we describe steps to visualize and quantify the biomass of these biofilms via crystal violet staining. Furthermore, we provide instructions how to stain and quantify polysaccharides in the extracellular biofilm matrix secreted by mycobacteria. Finally, we illustrate how to prepare both planktonic mycobacteria as well as biofilms on ALI-PBEC cultured in a BSL-3 environment for scanning electron microscopy (SEM). With these techniques combined, biofilms of various mycobacterial species can be studied in detail. While the host surface used here for biofilm formation is the bronchial epithelium, these techniques may be transferrable to epithelial cultures from other regions, such as alveolar or nasal epithelium, as well.

For detailed protocols to culture mycobacteria and ALI-PBEC, please refer to our related protocol in this issue of *STAR Protocols*, Barclay et al. (Barclay et al. co-submission 25-00065, this issue) and Ninaber et al.^4^

### Preparation of 7H9 bacterial culture medium

**Timing: 1 h**

1. Prepare the 7H9 broth.
  a. Weigh 4.7 g 7H9 broth powder stock and add to a 1 L glass bottle.
  b. Add 900 mL Milli-Q water, 2 mL glycerol and 0.5 mL Tween-80 (final concentrations 0.2% and 0.05%, respectively).
  c. Mix well by inverting the solution a few times.
  d. Sterilize the solution using an autoclave and allow to cool down to 45°C before proceeding.
2. During step 1d, prepare Middlebrook ADC supplement.
  a. Dissolve 50 g BSA, 20 g dextrose and 0.03 g catalase in 1 L Milli-Q water.
  b. Sterilize the solution using a 0.2 μm cellulose membrane syringe filter (Corning). Sterilized ADC supplement can be stored at 2°C–8°C up to 3 months.
3. Prepare 7H9 medium.
  a. Add 100 mL ADC supplement to the 7H9 broth from step 1 (final concentration 10%).
4. 7H9 medium must be stored at 2°C–8°C and used within 2 months.

### Preparation of 7H10 agar

**Timing: 2 h**

5. Prepare the 7H10 agar.
  a. Weigh 19 g 7H10 agar and add to a 1 L glass bottle.
  b. Add 900 mL Milli-Q water and 5 mL glycerol (final concentration 0.5%).
  c. Mix well by inverting the solution a few times.
  d. Sterilize the solution using an autoclave and let it cool down to 45°C before proceeding.
6. Meanwhile, put the Middlebrook OADC supplement (Thermo Fisher Scientific) at 18°C–21°C.
7. Once the 7H10 agar has cooled, add 100 mL OADC supplement.
8. Add the 7H10 agar to sterile 10 × 10 cm square petri dishes.
  a. Pipet 35 mL 7H10 agar per petri dish. Approximately 28 petri dishes can be made using 1 L agar.
  b. Allow the 7H10 agar to set at least 12 h at 18°C–21°C.
9. 7H10 agar plates must be stored at 2°C–8°C and used within 2 months.

### Preparation of cBD medium for epithelial cell culture

**Timing: 30 min**

The cBD epithelial cell culture medium consists of equal parts Dulbecco’s Modified Eagle’s Medium (DMEM) and Bronchial Epithelial Cell Medium-basal (BEpiCM) medium, supplemented with penicillin/streptomycin, HEPES, Glutamax and bronchial epithelial cell growth supplement (BEpiCGS).

**CRITICAL:** Prepare the cBD medium in a tissue culture flow cabinet to maintain sterility of the medium.

10. Add 5 mL penicillin/streptomycin (100x, final concentration 1x) to 500 mL of BEpiCM-b.
11. Add 5 mL penicillin/streptomycin (100x, final concentration 1x), 12.5 mL HEPES (1 M, final concentration 25 mM) and 5 mL Glutamax (100x, final concentration 1x) to 500 mL DMEM.
12. In a sterile 500 mL bottle, mix 250 mL BEpiCM-b prepared in step 10 and 250 mL DMEM prepared in step 11.
13. Add 5 mL of BEpiCGS (final concentration 1%) to the mixture prepared in step 12.
14. Aliquot 40 mL portions of the medium prepared in step 13 in 50 mL centrifuge tubes. The medium can be stored for 6 months at −20°C or at 4°C for 4 weeks.

***Note:*** To prepare antibiotics-free cBD medium, skip step 10, and do not add penicillin/streptomycin to DMEM during step 11. Then continue with the protocol from step 12.

### Preparation of paraformaldehyde-gluteraldehyde fixative for electron microscopy

**Timing: 30 min**

15. Prepare 0.4 M cacodylic acid.
  a. Add 950 mL of Milli-Q water to a 1 L glass bottle.
  b. Weigh 86 grams of cacodylic acid and add it to the bottle, mix well.
  c. Adjust the pH of the solution to 7.4 using hydrochloric acid and a pH meter.
  d. Add Milli-Q water until the total volume of the solution is 1 L. Store the solution at 4°C up to 6 months.
16. Prepare 0.2 M cacodylic acid buffer. Preparing the fixative with this buffer will help ultrastructural preservation during fixation.
  a. Add 5 mL of 0.4 M cacodylic acid prepared in step 15 to a 50 mL centrifuge tube.
  b. Add 5 mL of Milli-Q water to the tube and mix well.
17. Add paraformaldehyde (PFA) to the cacodylic acid buffer.
  a. Snap the lid of an ampule containing 16% PFA.
  b. Add 5 mL 16% PFA to the tube from step 16 (final concentration 4%) and mix well.
18. Add glutaraldehyde (GA) to the cacodylic acid buffer.
  a. Snap the lid of the ampule containing 8% GA.
  b. Add 5 mL 8% GA to the tube from step 16 (final concentration 2%) already containing the paraformaldehyde and mix well.
19. Close the tube and seal with parafilm. Fixative can be stored at 4°C up to 1 week. It is preferable to prepare fresh fixative shortly before performing experiments.

***Note:*** Cacodylic acid, 16% PFA and 8% GA are hazardous chemicals. Always wear protective gloves while preparing the fixative. Prepare the fixative in a fume hood and use the fixative in a flow cabinet.

### Preparation of storage solution for electron microscopy

**Timing: 20 min**

20. Prepare 0.1 M cacodylic acid buffer.
  a. Add 5 mL of 0.4 M cacodylic acid (prepared in step 15) to a 50 mL centrifuge tube.
  b. Add 14.38 mL Milli-Q water to the tube and mix well.
21. Add paraformaldehyde (PFA) to the cacodylic acid buffer.
  a. Snap the lid of an ampule containing 16% PFA.
  b. Add 0.62 mL 16% PFA to the tube from step 20 and mix.
22. Close the tube and seal the lid with parafilm around the edges. Storage solution can be stored at 4°C up to 6 months. It does not have to be prepared freshly for every experiment.

### Institutional permissions

Approval from your local ethics committee is required to perform experiments using patient-derived tissues or cells. Bronchial epithelial cells were isolated from lung tissue that appeared normal on a macroscopic level, obtained from patients undergoing resection surgery for lung cancer at the Leiden University Medical Center (LUMC), the Netherlands. Patients from whom this lung tissue was derived were enrolled in the biobank via a no-objection system for coded anonymous further use of such tissue (www.coreon.org). Samples from this Biobank were approved for research use by the institutional medical ethical committee (BB22.006/AB/ab). Since 01-09-2022, patients are enrolled in the biobank using written informed consent in accordance with local regulations from the LUMC biobank with approval by the institutional medical ethical committee (B20.042/KB/kb). Local regulations regarding biosafety may apply when working with pathogenic mycobacteria. Refer to your local regulations and permits to determine what containment level is appropriate for your strains.

## Key resources table

**Table:** 

REAGENT or RESOURCE	SOURCE	IDENTIFIER
**Antibodies**		

Human EpCAM/TROP-1 antibody produced in goat (used 20 times diluted)	R&D Systems	AF960
Donkey-anti-goat IgG (H+L) highly cross-adsorbed secondary antibody Alexa Fluor 405 (used 200 times diluted)	Thermo Fisher Scientific	A48259

**Biological samples**		

Primary human bronchial epithelial cells	Bronchial epithelial cells were isolated from lung tissue obtained from patients undergoing resection surgery for lung cancer at the Leiden University Medical Center (LUMC), the Netherlands. Samples were approved for research use by the institutional medical ethical committee (BB22.006/AB/ab and B20.042/KB/kb).	N/A

**Chemicals, peptides, and recombinant proteins**		

ProLong Diamond antifade mountant	Thermo Fisher Scientific	P36965
Wheat germ agglutinin stain conjugate Alexa Fluor 633	Thermo Fisher Scientific	W21404
Dulbecco’s modified Eagle’s medium (DMEM) + 4,500 mg/L D-glucose	STEMCELL Technologies	36250
Bronchial epithelial cell medium-basal (BEpiCM-b)	ScienCell Research Laboratories	SCC3211-b
Bronchial epithelial cell growth supplement (BEpiCGS)	ScienCell Research Laboratories	SCC3262
HEPES (1 M)	ScienCell Research Laboratories	600212-500
GlutaMAX	Thermo Fisher Scientific	35050038
EC 23 synthetic retinoid acid receptor agonist	Tocris Bioscience	4011
Penicillin/streptomycin (10,000 U/mL/10,000 μg/mL)	Thermo Fisher Scientific	15140122
Bovine albumin fraction V (BSA, 7.5% solution)	Thermo Fisher Scientific	15260037
Dextrose	Sigma-Aldrich	D9434
Catalase from bovine liver	Sigma-Aldrich	C1345-10G
BD Difco dehydrated culture media: Middlebrook 7H9 broth	Thermo Fisher Scientific	11753473
BD Difco dehydrated culture media: Middlebrook 7H10 agar	Thermo Fisher Scientific	11799042
BD BBL Middlebrook OADC enrichment	Thermo Fisher Scientific	17434923
Glycerol	Sigma-Aldrich	G9012
Tween 80	Sigma-Aldrich	P1754
Cacodylic acid sodium salt trihydrate, 98% pure	Thermo Fisher Scientific	214975000
Glutaraldehyde 8%, EM grade	Electron Microscopy Sciences	16019
Paraformaldehyde 16% solution, EM grade	Electron Microscopy Sciences	15710
Ethanol	VWR	83813.360
1,1,1,3,3,3-Hexamethyldisilazane	Carl Roth	3840.4
1% crystal violet solution	Sigma-Aldrich	V5265

**Other**		

SEM pin stubs	Agar Scientific	G301F
Conductive adhesive tabs	Electron Microscopy Sciences	76762-01
ACLAR embedding film	Electron Microscopy Sciences	50426-25
K650X sputter coater	Quorum Technologies (formerly Emitech)	K650X
Cellulose membrane (surfactant-free with prefilter) syringe filters with 0.2 μm pore size, used to filter sterilize medium components	Corning	CLS431218

## Materials and equipment

**Table:** 7H9 bacterial culture medium

Reagent	Final concentration	Amount
Milli-Q water	N/A	900 mL
7H9 broth	0.47%	4.7 g
Glycerol	0.2%	2 mL
Tween-80	0.05%	0.5 mL
Middlebrook ADC supplement (750 mM BSA + 110 mM dextrose + 130 μM catalase in Milli-Q water)	10%	100 mL
Total	N/A	1 L

Store at 18°C–21°C for 2 weeks or at 4°C and use within 2 months.

**Table:** 7H10 agar

Reagent	Final concentration	Amount
Milli-Q water	N/A	900 mL
7H10 agar	1.9%	19 gr
Glycerol	0.5%	5 mL
Middlebrook OADC supplement	10%	100 mL
Total	N/A	1 L

Use approximately 35 mL of agar per petri dish. Store at 4°C and use within 2 months.

**Table:** cBD epithelial cell culture medium

Reagent	Final concentration	Amount
BEpiCM-b with supplements	45%	250 mL
Penicillin/streptomycin (100x)	1x	2.5 mL
DMEM with supplements	45%	250 mL
Penicillin/streptomycin (100x)	1x	2.5 mL
HEPES (1 M)	25 mM	6.25 mL
Glutamax (100 x)	1x	2.5 mL
BEpiCGS	10%	5 mL
Total	N/A	500 mL

Store up to 6 months at −20°C or at 4°C up to 4 weeks.

**Table:** PFA-GA scanning electron microscopy fixative

Reagent	Final concentration	Amount
Milli-Q water	N/A	5 mL
Glutaraldehyde 8%	2%	5 mL
Paraformaldehyde 16%	4%	5 mL
0.4 M cacodylic acid, pH 7.4	0.1 M	5 mL
Total	N/A	20 mL

Store up to 1 week at 4°C.

**Table:** Electron microscopy storage solution

Reagent	Final concentration	Amount
Milli-Q water	N/A	14.38 mL
Paraformaldehyde 16%	0.5%	0.62 mL
0.4 M cacodylic acid, pH 7.4	0.1 M	5 mL
Total	N/A	20 mL

Store up to 6 months at 4°C.

## Step-by-step method details

### Part 1: Formation of biofilms on ALI-PBEC

**Timing: 1 h**

In this section, well-differentiated primary bronchial epithelial cells cultured at air-liquid interface (ALI-PBEC) are infected with mycobacteria to form biofilms on the cell layer. We refer to our related protocol (Barclay et al. co-submission 25-00065, this issue) and Ninaber et al.^4^ for detailed information on the culturing of ALI-PBEC and mycobacteria.

***Note:*** All procedures described in the following protocols are performed in biological safety cabinets under sterile working conditions, while wearing personal protective equipment in accordance with biosafety level 3 (BSL-3) regulations. BSL-3 containment is required for virulent Mtb strains. BSL-2 containment is often sufficient for less virulent species like *M. avium* or *M. bovis* (BCG). Refer to your local regulations and permits to determine what biosafety level is appropriate for your strains.

**CRITICAL:** All incubation steps that include epithelial cells should be performed at 37°C in a humidified incubator with 5% CO_2_. ALI-PBEC should be cultured in cBD medium without antibiotics during infections, and at least 48 h prior to infection.

1. Refresh medium of ALI-PBEC cultures previously prepared as described in our related protocol (Barclay et al. co-submission 25-00065, this issue) (perform until step 20) before infection.
  a. Remove cBD medium from the basal compartment of the wells.
  b. Add 1 mL warm (37°C) antibiotics-free cBD medium supplemented with 50 nM EC 23 to each well.

***Note:*** Evaluate the quality and differentiation of the PBEC cell layer prior to infection using an inverted microscope. The cell layer should be confluent, without gaps. If cell differentiation was successful, ciliated cells with beating cilia can be observed. In addition, the presence of gel-like mucus is an indicator of goblet cell differentiation. Trans-epithelial electrical resistance (TEER) can also be measured for quality control of the cultures. These analyses have been described in detail by Ninaber et al.^4^

***Note:*** We have previously determined that after two weeks of differentiation, our ALI-PBEC cultures contain on average 10^6^ cells. This can be determined by preparing single-cell suspensions (as described in our related protocol (Barclay et al. co-submission 25-00065, this issue), step 31–34) and counting cells manually using counting chambers, or with an automated cell counter.

2. Prepare bacterial suspensions in the desired multiplicity of infection (MOI), i.e. the ratio of bacteria to epithelial cells. Refer to our related protocol (Barclay et al. co-submission 25-00065, this issue) for instructions on culturing mycobacteria (perform until step 9).
  a. Dry 7H10 agar plates in a dry incubator at 37°C for at least 1 h before plating bacterial suspensions in step 3.
  b. Lightly shake mid-log bacterial cultures to homogenize. For each bacterial strain, prepare three dilutions in cuvettes: undiluted, diluted two times, and diluted five times in 7H9 medium.
  c. Measure OD_600_ of the strains.
  d. Calculate the number of bacteria per mL of culture by correlating the OD_600_ to CFU/ml to obtain the OD factor.
  e. Calculate the number of bacteria needed to obtain MOI 100 (100 bacteria per epithelial cell) and how much to dilute or concentrate the bacterial culture. An example is listed in Table 1.

**Table 1:** Example of MOI calculations

Dilution	OD_600_	Undiluted OD_600_	Average OD_600_	OD factor	Bacteria / mL	MOI 100 required bacteria / mL	Dilution factor	mL of culture	Resuspend in (mL)
1	0.600	0.600	0.660	1.3 x 10^7^	8.5 x 10^7^	2 x 10^9^	0.043	5	0.215
1:2	0.340	0.680							
1:5	0.140	0.700							
Calculations					= Avg. OD x OD factor	= 10^6^ cells^a^ x 100 x 20^b^	= $\frac{req.\;bact.}{bact.\;per\;ml}$		= dilution factor x ml of culture

a On average, an insert carries 10^6^ epithelial cells after two weeks of mucociliary differentiation.

b 50 μL volume is used per ALI-PBEC culture. Each mL contains 20 x 50 μL.
  f. Collect the required amount of bacterial culture in 15 mL or 50 mL centrifuge tubes and centrifuge for 10 min at 805 x g. Carefully remove supernatant without disturbing the pellet.
  g. Resuspend bacteria in the appropriate amount of antibiotics-free cBD supplemented with 50 nM EC 23 (Table 1) to reach the desired MOI.

***Note:*** The OD factor differs per bacterial strain, and can be calculated using methods detailed by Penuelas-Urquides et al.^5^

***Note:*** Mycobacteria are slow-growing species. Therefore, to initiate biofilm formation immediately, we routinely use MOI 100 to infect PBEC with a high load of bacteria. At this MOI, mycobacteria readily aggregate and enter the first stage of biofilm formation within 24 h.

**CRITICAL:** The mycobacterial cultures should be visually homogeneous to achieve accurate OD_600_ measurements. Cultures with clumps cannot provide reliable estimates of the desired multiplicity of infection. Mycobacteria should be in log phase (OD_600_ 0.2–1.0) at the time of preparing bacterial suspensions, to ensure optimal growth conditions.

3. Plate the bacterial suspensions on 7H10 agar to be able to retrospectively determine the accuracy of the prepared MOI. This is helpful to maintain consistency between experiments.
  a. Make 1:10 serial dilutions in PBS. The number of dilutions depends on the chosen MOI (example for MOI 100 suspension is included in Table 2).

**Table 2:** Serial dilutions to retrospectively determine actual MOI

Dilution	undiluted	10^–1^	10^–2^	10^–3^	10^–4^	10^–5^	10^–6^	10^–7^
**Expected no. of bacteria**	2 x 10^7^	2 x 10^6^	2 x 10^5^	2 x 10^4^	2000	200	20	2
  b. Plate 10 μL drops of each dilution on the dried 7H10 agar plates in triplicate.
  c. Allow the drops to absorb and dry, then seal the plate with tape along the edges to prevent dehydration of the agar during incubation.
  d. Incubate 7H10 plates in a dry incubator at 37°C.
  e. Check bacterial growth frequently and take plates out of the incubator when colonies are visible. The incubation time can vary between species. For example, 3–5 days for *M. smegmatis*, 10 to 15 days for *M. avium* and *M. bovis* and 14–21 days for *M. tuberculosis.*
    ***Note:*** To maintain consistency between experiments, an acceptable range of variation in MOI may need to be determined. For example, if aiming for MOI 100, experiments with MOI 20–200 may be accepted while experiments falling outside of this range may be disregarded.
    **CRITICAL:** Right before adding bacteria, wash the apical side of ALI-PBEC cultures to remove the mucus layer.
  f. Add 200 μL warm (37°C) PBS on top of each insert.
  g. Incubate inserts 10–15 min at 37°C.
  h. Carefully remove PBS with a pipette without touching the insert.
4. Add 50 μL bacterial suspension to the apical side of the inserts. Do not add bacteria to the basal compartment.

***Note:*** We previously determined that 50 μL is the optimal infection volume when using 12-well plate inserts of 1.13 cm^2^. Using this volume, bacteria can spread evenly over the insert, and ALI-PBEC mucociliary function and cell viability are not reduced. Uninfected control cultures should receive the same volume of antibiotics-free cBD medium supplemented with 50 nM EC 23.

**CRITICAL:** Centrifuge the plates with infected ALI-PBEC cultures for 2 min at 300 x g to spin down the bacteria onto the cells.

***Note:*** Centrifuging directly after adding bacterial suspension to ALI-PBEC brings the bacteria in direct contact with the cells before the epithelium secretes a substantial new mucus layer.

5. Incubate ALI-PBEC cultures at 37°C for 24 h or 7 days. Medium should be replaced every 2–3 days during this incubation. The apical surface should not be washed, as this will disturb biofilm formation.

***Note:*** The time frames of 24 h and 7 day incubation yield less developed and more developed biofilms, respectively. Incubation times may be increased further, although epithelial cell viability should be monitored, for example via microscopy.

### Part 2: Quantification of biofilm biomass by crystal violet staining

**Timing: 1 h**

**Figure 1:**
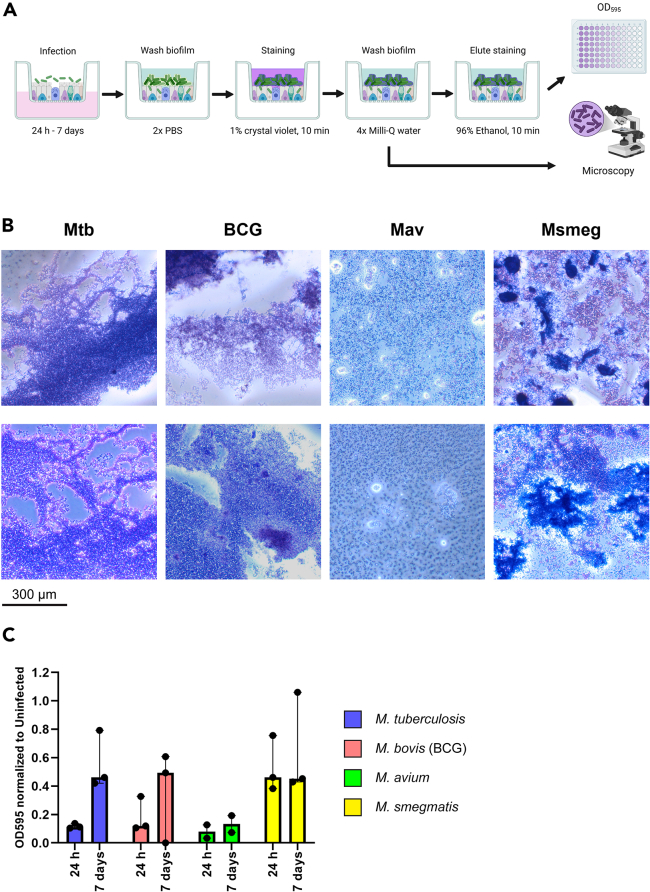
Crystal violet staining procedure and expected results (A) Schematic presentation of staining and washing steps in the crystal violet assay. Figure created in BioRender.com. (B) Examples of crystal violet-stained 7-day biofilms of Mtb, BCG and Msmeg. The Mav strain did not form proper biofilms within 7 days. Scale bar indicates 300 μm. (C) Bar graphs of optical density of crystal violet staining obtained from biofilms of four mycobacterial species from n= 2–3 independent ALI-PBEC donor mixes. Abbreviations: Mtb (*M. tuberculosis*), BCG (*M. bovis* Bacille Calmette-Guerin), Mav (*M. avium*), Msmeg (*M. smegmatis*). Data are shown as median with range. Data from experiments as published in Barclay et al.,^1^ unique images showing other fields of view not previously published.

In the next parts of the protocol, we describe several different methods to visualize and quantify biofilms formed on ALI-PBEC. Biomass of biofilms can be determined using crystal violet staining. Crystal violet stains mycobacteria and the extracellular matrix they produce. The optical density of the crystal violet staining is used to determine the total biomass of biofilms (Figure 1A). This staining should be performed on unfixed samples, at an appropriate biosafety level laboratory by trained staff following the local biosafety level restrictions. After staining, samples may subsequently be fixed for further microscopy analysis.

**CRITICAL:** When staining biofilms attached to cells, the epithelial cells and the mucus produced are (partially) stained as well. Therefore, it is important to include uninfected control PBEC cultures for later background staining subtraction.

***Note:*** We normally use at least 3 technical replicates, i.e. separate ALI-PBEC wells of the same donor, per condition to reduce potential variation within this assay.

6. After 24 h or 7 days of biofilm formation, remove bacterial suspensions from the apical side of ALI-PBEC, and the medium from the wells below inserts. Discard both medium and bacterial suspension.
7. Wash ALI-PBEC apically to wash away non-adherent bacteria.
  a. Pipet 200 μL PBS at 18°C–21°C on the insert by placing the pipette against the side of the insert. Slowly allow the liquid to enter the apical side of the cells via the wall of the insert.
  b. Carefully remove supernatant from the inserts. Do not disturb the biofilms with the pipette tip.
  c. Repeat this washing step once more.

***Note:*** Mycobacterial biofilms, particularly those that are incubated for only 24 h, can be fragile. Be extremely careful while washing biofilms to prevent parts of the biofilm detaching from the cells.

8. Stain adherent bacteria with crystal violet.
  a. Carefully add 200 μL of 1% crystal violet solution (Sigma-Aldrich) directly onto the cells of each insert, drop by drop. Do not pipette via the wall of the insert, as this may leave a residue that could interfere with the assay in step 10.
  b. Incubate for 10 min at 18°C–21°C.
  c. Remove crystal violet solution from each insert.
9. Wash excess crystal violet solution from biofilms.
  a. Add 200 μL Milli-Q water to each insert by placing the pipette against the side of the insert.
  b. Carefully remove water with a pipette without disturbing biofilms.
  c. Repeat this washing step three times.
10. Dissolve the crystal violet staining in ethanol.
  a. Add 200 μL 96% ethanol to each insert with a pipette.
  b. Incubate 10 min at 18°C–21°C.
  c. Transfer 100 μL from each insert to a clear 96-wells flat bottom plate.
  d. Prepare a 1:10 dilution of all samples in 96% ethanol.

***Note:*** Optical density of undiluted samples is likely too high (> 2.0) to accurately measure due to PBEC also being stained, although this depends on the detection range of the spectrophotometer used. Using a 1:10 or 1:100 dilution to measure optical density is required in this case.

11. Measure optical density at 595 nm.
12. Analyze results.
  a. Average OD_595_ values of technical replicates of uninfected control cultures.
  b. Subtract average uninfected control OD_595_ from all sample OD_595_ values.
  c. Multiply values by the dilution factor of the samples.

***Optional:*** Instead of dissolving and eluting crystal violet from the biofilm, stained biofilms can also be directly imaged using a light microscope. This technique works only for biofilms grown on transparent cell culture plates and not on biotic surfaces like PBEC, as crystal violet stains PBEC as well. To achieve this, perform the protocol until step 9c, and immediately image stained biofilms at 40–100 times magnification. Figure 1B shows examples of crystal violet-stained 7-day biofilms, and Figure 1C shows optical density of dissolved crystal violet staining.

### Part 3: Staining and quantification of biofilm matrix polysaccharides

**Timing: 3 days**

In this section, polysaccharides in the biofilm matrix on ALI-PBEC are stained and quantified to determine if polysaccharides are components of the matrix, and to study differences in biofilm matrix formation by different mycobacterial species. This protocol is an addition to our related protocol, Barclay et al. (Barclay et al. co-submission 25-00065, this issue). For this protocol, it is important to use fluorescently labeled mycobacteria to develop biofilms. We routinely use green fluorescent strains, and therefore use polysaccharide and membrane stains in the red and blue range of the fluorescent spectrum. When choosing fluorescent labels, ensure that there is little overlap in their spectrums, as this would complicate imaging.

***Note:*** Polysaccharides are stained with wheat germ agglutinin (WGA) and epithelial cell membranes are stained with EpCAM. WGA stains all types of sialylated polysaccharides, including those produced by epithelial cells and biofilms. Therefore, it is of importance to include a membrane stain to determine if polysaccharides overlap with epithelial cells or with bacterial fluorescence.

13. Fix infected inserts with 4% PFA.
  a. Wash inserts with PBS (0.5 mL apical side, 1.5 mL basal side) to remove traces of mucus and medium.
  b. Add 500 μL PFA apically and 1 mL basally.
  c. Seal the plate with tape along the edges.
  d. Incubate at least 24 h at 4°C.
14. Remove fixative.
  a. Wash inserts once with PBS, 500 μL apically and 1 mL basally.
  b. Add new PBS to each insert and well, 500 μL apically and 1 mL basally.
  c. Store cultures at 4°C in PBS and use within 6 months or proceed immediately with fluorescent staining.

***Note:*** After 24 h fixation, samples containing Mtb can be transferred to BSL-1 for further handling. If storing fixed inserts for a long time, regularly check if both sides of the insert are submerged in PBS. Do not let the cells dry out, as this will affect the quality of the staining.

15. Block the inserts with blocking buffer.
  a. Wash inserts once with PBS (500 μL apical, 1 mL basal).
  b. Add 500 μL blocking buffer (1% BSA, 0.3% Triton-X-100 solution in PBS) apically and 1 mL basally to each insert.
  c. Incubate 10 min at 18°C–21°C or 30 min at 4°C.

**Figure 2:**
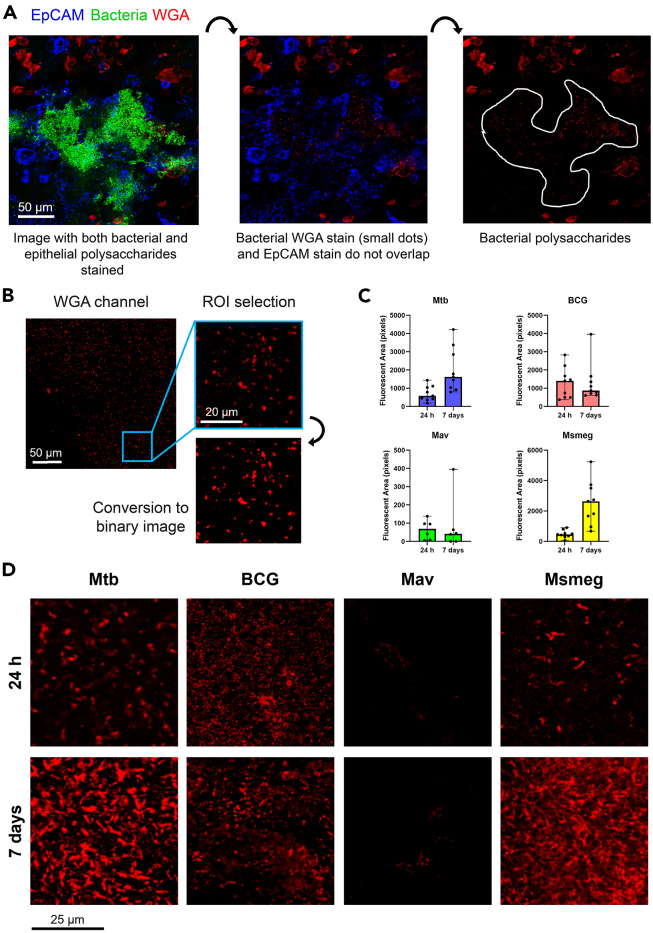
Quantification of polysaccharide staining and expected results (A) Method to differentiate between bacterial and epithelial polysaccharides. This is an image in the z-stack where biofilm and epithelial cell layer connect, and therefore it should be carefully determined whether polysaccharides are of bacterial or epithelial origin. Scale bar indicates 50 μm. (B) Example of the fluorescent channel corresponding to wheat germ agglutinin (WGA) staining in an Mtb biofilm, the region of interest (ROI) selected from this image, and ROI conversion to a binary image. Scale bars indicate 50 μm and 20 μm. (C) Bar graphs showing fluorescent area of WGA in pixels of 24 h and 7-day biofilms of four mycobacterial species. Data are shown as median with range. (D) Representative images of polysaccharide stains in 24 h and 7-day biofilms of four mycobacterial species. Abbreviations: Mtb (*M. tuberculosis*), BCG (*M. bovis* Bacille Calmette-Guerin), Mav (*M. avium*), Msmeg (*M. smegmatis*). Scale bar indicates 25 μm. Data from Barclay et al.^1^

***Note:*** For visual instructions for the following staining steps, we refer to Figure 2 in our related protocol Barclay et al. (Barclay et al. co-submission 25-00065, this issue).

16. Prepare primary EpCAM antibody solution.
  a. Cut out a piece of parafilm and fit it inside a lightproof plastic box.
  b. Dilute EpCAM primary antibody in blocking buffer to a concentration of 10 μg/mL (20 times dilution from 200 μg/mL stock).
  c. Add 50 μL drops containing primary antibodies on the parafilm.
17. Cut out the membrane from the inserts. Refer to Figure 2A Barclay et al. (Barclay et al. co-submission 25-00065, this issue).
  a. Invert the insert.
  b. Carefully slice around the edges of the membrane with a scalpel.
18. Incubate membranes with primary antibodies. Refer to Figure 2B Barclay et al. (Barclay et al. co-submission 25-00065, this issue).
  a. Using fine tweezers, place membranes on the drops containing primary EpCAM antibodies with cells facing the liquid.
  b. Close the lid of the box.
  c. Incubate membranes for 1 h at 18°C–21°C or 12–16 h at 4°C.

***Note:*** For the EpCAM antibody used in this protocol, 12–16 h incubation at 4°C resulted in more specific antibody binding than incubation at 18°C–21°C. In addition, the tweezers used to handle the membrane may damage the cell layer if not used carefully. To minimize damage to the cell layer during handling steps, try to position the tweezers in the same area of the membrane each time.

19. Prepare secondary antibodies and wheat germ agglutinin (WGA) stain.
  a. Prepare a second box with parafilm as in step 16a.
  b. Dilute secondary donkey-anti-goat Alexa Fluor 405 antibody 200 times to a concentration of 10 μg/mL (from 2 mg/mL stock) in blocking buffer.
  c. To this antibody solution, add WGA 633 stain until a final concentration of 5 μg/mL (diluted 200 times from 1 mg stock).
  d. Add 50 μL drops of the solution to the parafilm in the box.
20. Wash excess primary antibody solution from the membranes. Refer to Figure 2C Barclay et al. (Barclay et al. co-submission 25-00065, this issue).
  a. Fill three wells of a 6-well culture plate with PBS.
  b. Carefully grab the edge of a membrane section with tweezers.
  c. Wash the membrane pieces three times by slowly swinging them in the PBS.
21. Incubate membranes with secondary antibody solution.
  a. After washing, immediately place membrane pieces with cells facing the drops containing secondary antibodies and stains. Immediately close the lid of the box.
  b. Incubate in the dark for 2 h at 4°C.
22. Wash excess secondary antibody solution from the membranes.
  a. Fill three wells of a 6-well plate with PBS and three wells with Milli-Q water.
  b. Wash membranes three times by dipping in PBS and swirling them around.
  c. Wash membranes three times by dipping in Milli-Q water and swirling them around.
23. Mount membranes on microscopy slides. Refer to Figures 2D and 2E Barclay et al. (Barclay et al. co-submission 25-00065, this issue).
  a. Lightly dab a corner of the membrane onto tissue paper to remove excess liquid.
  b. Place membrane on a glass microscopy slide with cells facing upwards.
  c. Add a drop of anti-fading reagent on top of each membrane piece.
  d. Cover membranes with a cover slip.
  e. Firmly press the cover slip to remove air bubbles trapped below the glass.
  f. Allow anti-fading reagent to set 12–16 h at 18°C–21°C.
  g. Store microscopy slides at 18°C–21°C in a dark dry place. Stained inserts treated with anti-fading reagent can be stored up to 3 months without loss of fluorescent signal.
24. Image membranes using a confocal fluorescence microscope. Expected results are shown in Figure 2. The microscope used to produce the images in this figure is a Leica SP8-WLL confocal microscope, with a 40x magnification oil immersion objective. Other similar microscopes may be used instead.

***Note:*** The PBEC cell layer and biofilm together are approximately 30 μm thick. We recommend taking images at 1 μm intervals throughout the whole sample when creating a z-stack.

***Note:*** WGA stains sialylated polysaccharides, including those expressed by PBEC. Therefore, ROI should be chosen carefully not to include epithelial cells. An example of bacterial and epithelial polysaccharides in the same image is given in Figure 2A. Bacterial polysaccharides should overlap with bacterial fluorescence, but not with EpCAM staining. This can be achieved by choosing an image in the z-stack that shows predominantly bacterial fluorescence and little to no EpCAM staining. The cell layer may be damaged in areas where biofilms are formed. Therefore, EpCAM signal may already co-localize less with biofilms. An example of an area where epithelial and bacterial signal overlap partially in a damaged section of the cell layer is shown in Figure 2A., biofilm-associated WGA staining can be distinguished from epithelial-associated staining by its distinct pattern of puncta. By imaging areas with biofilms at multiple planes using z-stacks, it is recommended to select planes where bacterial and epithelial fluorescence ideally do not overlap, or have very minimal overlap. From these images, 50 x 50 μm ROI can be selected (Figure 2B). As biofilms may vary in thickness and density in different locations on the same ALI-PBEC insert, we recommend selecting at least 3 ROI per insert.

25. Quantify the WGA staining.
  a. Using the software of the fluorescence microscope, select images from the z-stack containing predominantly bacterial fluorescence and little EpCAM fluorescence.
  b. Export the separate fluorescent channels as images.
  c. Select regions of interest (ROI) of 50 x 50 μm in the WGA channel for each biofilm.
  d. Import the image of the WGA channel in Fiji software.
  e. Convert the WGA channel to a binary state using the ‘Color Threshold’ option to differentiate between fluorescent and non-fluorescent pixels (Figure 2A).
  f. Calculate the sizes of all fluorescent areas using the ‘Analyze Particles’ option in ImageJ. Use a 3-pixel-minimum threshold per area to exclude background staining.
  g. Calculate total WGA fluorescent signal by summing up pixel sizes of all areas per ROI (Figure 2C). Examples of WGA stained biofilms of different species are shown in Figure 2D.

***Optional:*** The bacterial density in the biofilm can be estimated using the same method as described for WGA. In this case, use the fluorescent channel corresponding to the bacteria.

### Part 4: Visualization of biofilm ultrastructure by scanning electron microscopy

**Timing: 4 days**

With scanning electron microscopy (SEM), it is possible to visualize mycobacteria and mycobacterial biofilms and investigate their morphology at high magnifications. Here, we describe how to prepare biofilms on ALI-PBEC as well as planktonic bacteria for SEM. Fixation of mycobacteria should take place at the appropriate biosafety level, according to local regulations, after which the samples can be transferred to BSL-1.

26. Fix ALI-PBEC inserts containing biofilms.
  a. Remove medium from the wells.
  b. Carefully add 200 μL fixative (4% paraformaldehyde and 2% glutaraldehyde in 0.1 M cacodylic acid (PFA-GA) as prepared in the before you begin section, step 15–19) to the apical side of inserts without disturbing biofilms.
  c. Add 1 mL PFA-GA fixative to the wells below inserts.
  d. Incubate 30 min at 18°C–21°C.
  e. Incubate at least 24 h at 4°C.
27. Fix a suspension of 2 x 10^9^ bacteria per mL from liquid bacterial cultures (refer to our related protocol for instructions on culturing mycobacteria (Barclay et al. co-submission 25-00065, this issue, Part 1).
  a. Using the same method as described in step 2c-f of this protocol, prepare a pellet of 2 x 10^9^ bacteria in a 15 mL or 50 mL conical centrifuge tube.
  b. Resuspend the pellet in 5 mL PFA-GA fixative.
  c. Incubate 30 min at 18°C–21°C.
  d. Incubate 3 days at 4°C.

***Note:*** Ensure the fixative is at 18°C–21°C before applying to the inserts. Both liquid bacterial suspension and biofilms can now be transferred to BSL-1 level for further handling.

28. Remove fixative from inserts.
  a. Wash inserts once with 0.1 M cacodylic acid, pipetting 500 μL apically and 1 mL in the wells below inserts.
  b. Add storage solution (0.5% PFA in 0.1 M cacodylic acid, as prepared in the before you begin section, step 20–22), 500 μL apically and 1 mL in the wells.
  c. Store cultures at 4°C in storage solution until further processing, or proceed immediately with dehydration.

***Note:*** If storing fixed cultures on inserts for a long time (> 1 week), regularly check if both sides of the insert are submerged in storage solution. Do not let the cells dry out, as this will affect the quality of the morphology.

29. Prepare 0.1 M cacodylic acid.
  a. Add 5 mL of 0.4 M cacodylic acid (prepared in before you begin, step 15) to a 50 mL centrifuge tube.
  b. Add 15 mL of Milli-Q water to the tube and mix well.
30. Dehydrate the inserts.
  a. Wash inserts once with 0.1 M cacodylic acid (prepared in step 29): 500 μL apically and 1 mL basally.
  b. Add 500 μL 70% ethanol to the apical side of each insert and 1 mL in the wells.
  c. Incubate 30 min at 18°C–21°C, then remove 70% ethanol.
  d. Add 500 μL 90% ethanol to the apical side of each insert and 1 mL in the wells.
  e. Incubate 30 min at 18°C–21°C, then remove 90% ethanol.
  f. Add 500 μL 100% ethanol to the apical side of each insert and 1 mL in the wells.
  g. Incubate 30 min at 18°C–21°C, then remove 100% ethanol.
  h. Repeat step 30f-g once more, and continue with step 31a during the incubation time.

**CRITICAL:** After removing ethanol from an insert during sub-steps of step 30, immediately continue the protocol and add new ethanol (from the next sub-step) to that insert. Do not first remove ethanol from all the inserts before adding new ethanol, as the samples will dry out and this will affect the morphology.

**Figure 3:**
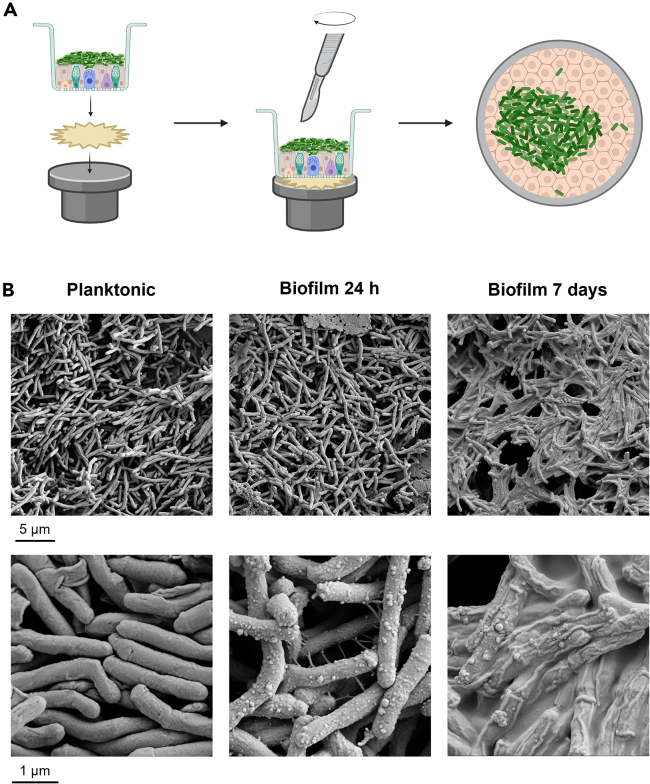
Visualization of biofilms and associated extracellular matrix by scanning electron microscopy (A) Schematic overview of the steps to mount ALI-PBEC inserts on SEM stubs. Figure created with BioRender.com. (B) Example of expected results from SEM. Bacteria shown are *M. smegmatis*. Magnification of top row of images is 25.000x and bottom row 150.000x. Top row: Scale bar indicates 5 μm. Bottom row: Scale bar indicates 1 μm. Images from Barclay et al.^1^

***Note:*** Do not remove the membrane from the insert before mounting. This will result in the membrane having an irregular surface, and will disrupt the dried biological material.

31. Dry the inserts.
  a. Mix equal amounts of 100% ethanol and 100% hexamethyldisilazane (Et-HMDS).
  b. Remove 100% ethanol from inserts (from step 30h).
  c. Add 500 μL Et-HMDS solution to the apical side of each insert and 1 mL in the wells.
  d. Incubate 30 min at 18°C–21°C, then remove Et-HMDS solution.
  e. Add 500 μL 100% HMDS apically and 1 mL in the wells.
  f. Incubate 30 min at 18°C–21°C, then remove 100% HMDS.
  g. Air-dry the inserts completely.
32. Mount membranes on SEM pin stubs (Agar Scientific) (Figure 3A).
  a. Mount a conductive adhesive tab on a SEM pin stub.
  b. Mount the insert with basal side of the membrane on the conductive adhesive tab, so the cell layer with biofilm is facing upwards.
  c. Use a scalpel to detach the membrane from the insert, and remove the empty plastic insert.

33. Coat the membranes with gold/palladium (Au/Pd) to produce a conductive layer on the samples. This will prevent electric charging of the samples during imaging, which gives rise to anomalous contrast in the SEM images.
  a. Place the SEM pin stubs with membranes in a sputter coater (Quorum Technologies).
  b. Metal coat the membranes using an Au/Pd target for 2.5 min with an electric current of 35 mA on the target. The coating chamber should reach a 0.2 mBar vacuum before coating.
  c. After coating, wait until the coating chamber has been purged with argon and pressure returns to baseline. Then remove pin stubs from the sputter coater.
  d. Store the SEM pin stubs with the membranes until imaging.

***Note:*** The protocol for preparing liquid bacterial cultures differs from the protocol for ALI-PBEC. Below, we detail the steps for liquid cultures. The following steps use the fixed liquid bacterial cultures from step 27.

34. Remove fixative from fixed liquid bacterial cultures from step 27.
  a. Centrifuge bacteria at 2576 x g for 3 min at 18°C–21°C.
  b. Wash bacteria once with 5 mL 0.1 M cacodylic acid.
  c. Centrifuge bacteria at 2576 x g for 3 min at 18°C–21°C.
  d. Resuspend bacteria in 5 mL 0.5% PFA in 0.1 M cacodylic acid.
  e. Store cultures at 4°C in 0.5% PFA in 0.1 M cacodylic acid until further processing, or proceed immediately with dehydration.
35. Dehydrate bacteria.
  a. Wash bacteria by adding 5 mL 0.1 M cacodylic acid.
  b. Centrifuge bacteria at 2576 x g for 3 min at 18°C–21°C, remove supernatant.
  c. Completely resuspend bacteria in 5 mL 70% ethanol and incubate 30 minutes at 18°C–21°C.
  d. Centrifuge bacteria at 2576 x g for 3 min at 18°C–21°C, remove the supernatant.
  e. Completely resuspend bacteria in 5 mL 90% ethanol and incubate 30 minutes at 18°C–21°C.
  f. Centrifuge bacteria at 2576 x g for 3 min at 18°C–21°C, remove the supernatant.
  g. Completely resuspend bacteria in 5 mL 100% ethanol and incubate 30 minutes at 18°C–21°C.
  h. Centrifuge bacteria at 2576 x g for 3 min at 18°C–21°C, remove supernatant.
  i. Repeat step 35g-h once more and continue with step 36a during the incubation time.

**CRITICAL:** After removing the ethanol from a pellet during sub-steps of step 35, immediately continue the protocol and add new ethanol (from the next sub-step). Do not first remove ethanol from all the pellets before resuspending in new ethanol as the samples will dry out, and this will affect the morphology.

36. Dry bacteria.
  a. Mix 2.5 mL of 100% ethanol and 2.5 mL of 100% hexamethyldisilazane (Et-HMDS).
  b. Centrifuge bacteria at 2576 x g for 3 min at 18°C–21°C, remove the supernatant.
  c. Resuspend bacteria in 5 mL Et-HMDS solution and incubate 30 minutes at 18°C–21°C.
  d. Centrifuge bacteria at 2576 x g for 3 min at 18°C–21°C, remove the supernatant.
  e. Resuspend bacteria in 5 mL 100% HMDS and incubate 30 minutes at 18°C–21°C.
  f. Centrifuge bacteria at 2576 x g for 3 min at 18°C–21°C, remove the supernatant.
  g. Resuspend the pellet in 50 μL of 100% HMDS.
  h. Prepare a disc of 1 cm in diameter from ACLAR film (Electron Microscopy Sciences).
  i. Spot approximately 50 μL of the bacterial suspension on the ACLAR film disc.
  j. Air dry the bacteria on the ACLAR film disc.
37. Mount bacteria on SEM pin stubs.
  a. Mount a conductive adhesive tab on a SEM pin stub.
  b. Mount the ACCLAR film disc with bacteria on the conductive adhesive tab, with bacteria facing upwards.
38. Coat the bacteria with gold/palladium (Au/Pd) to produce a conductive layer on the samples. This will prevent electric charging of the samples during imaging, which gives rise to anomalous contrast in the SEM images.
  a. Place the SEM pin stubs with the bacteria in a sputter coater (Quorum Technologies).
  b. Metal coat the bacteria, using an Au/Pd target for 2.5 min with an electric current of 35 mA on the target. The coating chamber should reach a 0.2 mBar vacuum before coating.
  c. After coating, wait until the coating chamber has been purged with argon and pressure returns to baseline. Then remove pin stubs from the sputter coater.
  d. Store the SEM pin stubs with the bacteria until imaging.
39. Image bacteria and ALI-PBEC membranes using a scanning electron microscope such as the Zeiss Gemini 300, operated at 5.0 kV. Examples of images obtained using this protocol are given in Figure 3B.

## Expected outcomes

This protocol was developed to establish, visualize and quantify mycobacterial biofilms on primary human bronchial epithelial cells. Using this protocol, biofilm formation can be successfully achieved on a biotic surface, although it must be noted that biofilm formation may differ per bacterial species, as some species are more efficient at forming aggregations, or they are faster-growing. In addition, as primary cells are used in this protocol, inter-donor variation may also play a role in the efficiency of biofilm formation. For example, some donors may have stronger mucociliary clearance or secretion of antimicrobial components than others. On average, with the species of mycobacteria that we have tested (Mtb, BCG, Mav and Msmeg), we achieve consistent biofilm formation within 24 h with all species except Mav. However, this may differ for other mycobacterial species and even between different strains of the same species.

## Limitations

A limitation of this procedure is that biofilms, particularly less developed biofilms at 24 h, are rather fragile and may detach easily from ALI-PBEC, which may hinder data collection. Furthermore, during the long incubation times required to obtain well-developed biofilms, contact with bacteria for a prolonged time may cause damage to the mucosal barrier. Therefore, integrity of the epithelial cells should be monitored closely. Finally, the composition of cell types in cultures of different donors may vary, which in turn can influence the formation of biofilms.

## Troubleshooting

### Problem 1

Mycobacteria are forming aggregates in liquid culture.

### Potential solution 1

Make sure that there is sufficient Tween-80 in the 7H9 broth. As Tween-80 is a viscous liquid, check that the correct amount is being pipetted during 7H9 medium preparation. Alternatively, split bacterial cultures to a lower OD_600_ the day before experiments, for example at OD_600_ = 0.125.

### Problem 2

Lack of access to BSL-3 facilities to culture Mtb.

### Potential solution 2

Non-virulent Mtb strains like H37Ra or other related species like BCG or Msmeg can be cultured at BSL-2 facilities and can be used as model organisms.

### Problem 3

Biofilms are detaching from ALI-PBEC during the crystal violet assay washing steps (step 7 and 9).

### Potential solution 3

Pipet the washing solutions extremely carefully and slowly by placing the pipette against the wall of the insert. Do not incubate biofilms in the washing solutions for more than one minute. Alternatively, reduce the number of washing steps.

### Problem 4

WGA staining is not successful (step 19).

### Potential solution 4

Increase the concentration of WGA to 10 μg/mL. Alternatively, your bacterial strain may produce fewer polysaccharides and it may be more useful to select a different marker to stain the biofilm matrix. For example, eDNA (which is present in most bacterial biofilms) could be stained by various methods including TOTO-1 stain.

### Problem 5

Charging occurs during scanning electron microscopy imaging (step 33 and 38).

### Potential solution 5

Metal coat the samples under different angles, to coat the bacteria everywhere. Using a rotating specimen table in the sputter coater (if available) is preferred, to create a more uniform coating. Also, it might be useful to make a connection between the bacteria and the SEM stub at the side of the stub with conductive paint, because the ACCLAR film might act as an insulator.

## Resource availability

### Lead contact

Further information and requests for resources and reagents should be directed to and will be fulfilled by the lead contact, Simone A. Joosten (s.a.joosten@lumc.nl).

### Technical contact

Technical questions on executing this protocol should be directed to and will be answered by the technical contact, Amy M. Barclay (a.m.de_waal@lumc.nl).

### Materials availability

This study did not generate new unique reagents.

### Data and code availability

All data presented are derived from the same experiments as reported in Barclay et al.^1^ Original crystal violet data, scanning electron microscopy images, and confocal microscopy quantification data have been deposited to Mendeley Data: https://data.mendeley.com/datasets/xvzh5y5dhg/1. Raw confocal microscopy data reported in this article will be shared by the lead contact upon request.

## Acknowledgments

This work was supported by a grant from the Leiden-Edinburgh Joint PhD program for Integrated One Health Solutions, awarded by the universities of Leiden and Edinburgh in December 2019. The graphical abstract for this publication was created with BioRender.com.

## Author contributions

A.M.B. optimized the infection, crystal violet, and confocal protocols; collected and analyzed data; and drafted the article. R.W.A.L.L. optimized scanning electron microscopy protocols to image biofilms on inserts as well as planktonic bacteria and performed all electron microscopy imaging work. M.B., P.S.H., T.H.M.O., A.M.v.d.D., and S.A.J. served as scientific advisors and critically reviewed the protocols, study design, and article.

## Declaration of interests

The authors declare no competing interests.
